# Health Tract, No. 103

**Published:** 1862-09

**Authors:** 


### "PRESERVES"

ARE sometimes deadly poisons, in consequence of the improper material of the vessels in which they are made or are contained. If made in copper or brass kettles, the utmost and closest attention should be given, to see that every spot the size of a pin should fairly glisten by vigorous and thorough scouring. But even this will not avail if the preserves themselves are imperfectly sweetened, or are not thoroughly cooked. A defect in either case will result in corroding the cans or jars in which they are put for keeping. This corrosion makes chemical combinations which are fatal to life, or lay the foundation for long, distressing, and obscure diseases. The only perfectly safe preserve-jar is that which is made of glass. All others ought to be discarded. They are cheap, more easily and more perfectly cleaned, and with reasonable care, will last a lifetime. And as every family with any claim to thrift, respectability, and hospitality, aims to have more or less of "preserves" for the winter months, the fact of having safe "preserves," as to health, is of very general interest; otherwise they are no "preserves" at all, but "destructives" alike to health and even life. It certainly would be better to have none at all, for they tempt us to eat when we do not feel much like eating, especially at tea-time, and thus aid in making many miserable dyspeptics; but as "thrifty housewives" will have them, it is well to instruct the public as to the best means of having them free from actual poison. It is to be hoped that no intelligent, conscientious person will keep preserves in any other vessel hereafter than those of glass. The jars should be made of "blown," not pressed glass, and if uniformly thin, are less liable to break by the fermentation of their contents.

But as external air will cause fermentation, every jar should be made perfectly air-tight. Cork alone can never do this, unless a trench is dug in a good cellar and the filled jars are put in, mouths down, and then well covered with earth or sand A better plan is that advised by the *Scientific American*. Waxed cloth tied over the jar is a substitute at once cheap and effective, and we have never found any thing superior to it. Prepare the cloth thus: melt together some rosin, beeswax, and tallow in equal parts; tear the cloth in strips four inches wide, or at least wide enough conveniently to tie over the mouth of the jar, and dip these strips, drawing them through the hot wax and stripping nearly all the wax off. With cloth thus prepared, after the jar is filled with hot preserves, and while still hot, close the mouth and bind it on with good linen cord. Then with shears trim off as much of the waxed cloth as is desirable, and then dip it in some melted wax, which should be made with only about half as much tallow. Sealing-wax may be used instead if desired. The jars should be put where the wax will cool at once, so that the exhaustion, caused by the cooling of the preserves and the condensation of the steam, may not cause the wax to run through the cloth. Nothing can be more thoroughly air-tight than bottles so prepared.

Self-sealing air-tight glass jars, which are now so common, are the best vessels for securing preserved fruits, but the above is good advice to those who have plenty of common glass jars and bottles.

